# Clinicopathological features and treatment outcome of juvenile idiopathic inflammatory myopathies with anti-melanoma differentiation associated gene 5 antibodies: A case series study

**DOI:** 10.1097/MD.0000000000039523

**Published:** 2024-08-30

**Authors:** Long Liu, Ying Hou, Dandan Zhao

**Affiliations:** a Department of Pathology, Qilu Hospital, Cheeloo College of Medicine, Shandong University, Jinan, Shandong, China; b Department of Neurology, Qilu Hospital of Shandong University, Jinan, Shandong, China; c Research Institute of Neuromuscular and Neurodegenerative Diseases and Department of Neurology, Qilu Hospital, Cheeloo College of Medicine, Shandong University, Jinan, China.

**Keywords:** anti-MDA5 antibodies, juvenile idiopathic inflammatory myopathies, MHC-I expression, treatment outcome

## Abstract

To characterize the clinicopathological features and treatment outcomes of juvenile idiopathic inflammatory myopathies (JIIM) with anti-melanoma differentiation associated gene 5 (MDA5) antibodies in a Chinese cohort. Anti-MDA5 antibody was detected by immunodot assay and indirect immunofluorescence assay on HEK293 cells in a series of Chinese JIIM cohort between 2005 and 2022. The clinical features, histological findings, and treatment outcomes of these anti-MDA5-antibody-positive patients were summarized. Of 59 JIIM patients, 3 (5.08%) were found to be anti-MDA5-antibody-positive. The frequency of anti-MDA5 antibody did not show significant difference between adult idiopathic inflammatory myopathies and JIIM cohorts (*P* = .720). The disease duration in patients with anti-MDA5 antibody was 2.83 ± 1.04 months. All 3 patients had typical skin lesions including Gottron sign and heliotrope rash, while interstitial lung disease and arthritis was only found in 1 patient. All 3 patients showed normal creatine kinase levels. On muscle biopsy, diffuse major histocompatibility complex class-I expression was seen in 3 patients and myxovirus-resistance protein A expression was found in 2 patients. All patients received long-term follow-up (6.42 ± 4.01 years). They were all drug-free and showed favorable treatment outcome with prednisone and additional immunosuppressant. Our study indicates that anti-MDA5 antibodies may not be common in Chinese JIIM. Anti-MDA5-positive JIIMs are characterized by typical skin lesions of dermatomyositis, normal CK levels, and increased major histocompatibility complex class-I expression. JIIMs with anti-MDA5 generally have good response to immunotherapies.

## 1. Introduction

Idiopathic inflammatory myopathies (IIMs) are a heterogeneous group of autoimmune disorders characterized by proximal muscle weakness and extramuscular involvement including skin lesion, interstitial lung disease (ILD) and arthritis.^[1]^ In particular, patients with onset age of first symptom ≤18 years are classified as juvenile idiopathic inflammatory myopathy (JIIM).^[2]^

Myositis-specific antibodies (MSAs) delineate distinct clinical subsets of IIM in both adult and juvenile patients, thus they hold important diagnostic and prognostic values.^[3,4]^ Anti-melanoma differentiation associated gene 5 (MDA5) was initially identified in adult IIM patients and associated with clinically amyopathic myositis, ILD and a poor prognosis, while the characteristics may be different between anti-MDA5-positive JIIMs and anti-MDA5-positive adult IIMs.^[5–7]^ Indeed, the prevalence of anti-MDA5 antibody in JIIM and the clinical features of these anti-MDA5-positive cases varies considerably in different ethnicities. Specifically, the prevalence of anti-MDA5 autoantibodies in JIIM is up to 40% in Japan, while <10% in Western countries and in India.^[5,8–13]^ ILD was regarded as a distinguished feature in anti-MDA5-positive JIIM patients in North America and Japan,^[9,10,14]^ while this finding is not confirmed in cohorts of UK and India.^[8,11]^ Besides, the expression of myxovirus-resistance protein A (MxA) also remains undetermined. Studies from Europe find no or weak MxA expression in patients with anti-MDA5 antibody,^[15,16]^ while all Japanese patients show MxA expression.^[17]^

In this study, we explored the prevalence of anti-MDA5 autoantibodies among 59 JIIM patients in Chinese cohort and summarized the clinical and histopathological characteristics as well as treatment outcomes of these 3 anti-MDA5-positive patients to improve the early diagnosis and the precise treatment of Chinese anti-MDA5-positive JIIM patients.

## 2. Materials and methods

### 2.1. Participants

We retrospectively reviewed clinical and histopathological data of 575 consecutive IIM patients in Department of Neurology, Qilu Hospital located in northern China between April 2005 and December 2022. The diagnosis of IIM was made based on the ENMC criteria as well as the EULAR/ACR criteria.^[2,18,19]^ Exclusion criteria including patients without myositis-specific antibody (MSA) screening or muscle biopsy and/or patients with clinical features of sporadic inclusion body myositis. Altogether, 499 out a total of 575 patients fulfilled the inclusion/exclusion criteria for the present study. Among them, 59 were defined as JIIM patients since the onset age of first symptom ≤18 years.^[2]^ Muscle biopsies were re-assessed by 2 IIM experts (YH and BZ). We reviewed their clinical manifestations, laboratory findings, muscle pathological features, and treatment regimens and outcomes.

In regard to clinical assessments, muscle strength was evaluated by the ordinal 6-point (0–5) manual muscle testing scale. The normal value of serum CK is ranged from 38 to 174 U/L. The normal value of C-reactive protein (CRP) is <6 mg/L. CK, a CRP were tested before muscle biopsy and prednisone treatment. Rapid progressive ILD (RP-ILD) was defined as progressive dyspnea or progression of high-resolution CT findings within 3 months of respiratory symptom onset or at the time of diagnosis of IIM.^[20]^ Treatment outcomes were graded as: no improvement, mild improvement (1 grade improvement in at least 1 muscle group, persistently requiring assistance in daily activities), moderate improvement (>1 grade in multiple muscle groups, occasionally requiring assistance in daily activities), marked improvement (only mild weakness without functional impairment) or returning to baseline (no symptoms or signs of muscle weakness); a favorable outcome was defined as marked improvement or returning to baseline.^[21]^

### 2.2. MSAs detection

Serum was available from 499 IIM patients. These samples were all stored at −80 °C prior to analysis and all of them were tested for MSAs and myositis-associated antibodies. In fact, we also recruited 100 healthy controls (HCs) for strengthening quality control since some novel MSAs, such as anti-Zo A antibody and anti-Zo B antibody, were not routinely tested. To address the different frequency of anti-MDA5 antibody in adult IIMs and in JIIM cohorts, anti-MDA 5 antibodies were measured in 499 patients with IIM including 440 adult IIMs and 59 JIIMs and 100 HCs including 85 adults and 15 juveniles. There was no significant difference on sex and age between IIM cohorts and HCs. The detection of anti-MDA 5 antibodies was made by immunodot assay (MT313-16, Autoimmune Myositis Profile Antibody IgG Detection Kit) and indirect immunofluorescence assay (IIFA). IIFA on HEK293 cells was made as following: human MDA5 coding sequence (NM_022168.4) was subcloned into pcDNA3.1 plasmid, and transfected into HEK293T cells. 48 hours later, cells were fixed with cold acetone. Fixed cells were incubated with patients’ serum (1:10 diluted with PBS) for 1 hour, then incubated with corresponding FITC labeled secondary antibodies (FITC-goat anti-human IgG Fcg antibody (109-095-170), Jackson ImmunoResearch, PA) for IgG detection. Immunostained cells were screened with fluorescence microscope (EVOS M5000, Thermo Fisher Scientific Inc, Carlsbad, CA).

In addition, anti-Mi2α antibody, anti-Mi-2β antibody, anti-TIF1γ antibody, anti-NXP2 antibody, anti-SAE antibody, anti-SRP antibody, anti-HMGCR antibody, anti-Jo-1 antibody, anti-Zo A antibody, anti-Zo B antibody, anti-PL7, antibody, anti-PL12 antibody, anti-EJ antibody, anti-OJ antibody, anti-cN1A antibody, anti-Ku antibody, anti-PM-Scl100 antibody, anti-PM-Scl75 antibody, and anti-Ro52 antibody were also tested by immunodot assay (MT313-16, Autoimmune Myositis Profile Antibody IgG Detection Kit).

### 2.3. Muscle biopsy

Muscle biopsies were taken from biceps brachii, deltoid or quadriceps of all 59 patients with JIIM. Serial frozen sections were stained with hematoxylin and eosin, anti-CD3 mouse monoclonal antibody (clone LN10; Zhongshan Golden Bridge Biotechnology, China), anti-CD8 rabbit monoclonal antibody (clone SP16; Zhongshan Golden Bridge Biotechnology), anti-major histocompatibility complex class-I (MHC-I) rabbit monoclonal antibody (clone EP1395Y; Abcam, UK), anti-major histocompatibility complex class-II (MHC-II) mouse monoclonal antibody (clone CR3/43; Dako, Denmark), anti-membrane attack complex (MAC) mouse monoclonal antibody (clone aE11; Dako), and anti-MxA rabbit polyclonal antibody (Abcam). The sarcoplasmic MxA expression was defined as the cytoplasmic positivity of MxA in non-necrotic fibers.^[22]^ Perifascicular (PF) pattern was defined as positive immunostaining limited to the peripheral area of muscle fascicles.

This study was approved by the ethics committee of Qilu Hospital (KYLL-202011-135). All methods were performed in accordance with the relevant guidelines and regulations. The guardian of all patients gave their informed written consent.

### 2.4. Statistical analysis

Qualitative variables were expressed as percentages and absolute frequencies, while quantitative features were reported as mean and standard deviation values. Categorical variables were analyzed by Fisher exact test as predicted frequency <5. Statistical significance was defined as *P* < .05. All analyses were performed using SPSS 22.0 (IBM, NY).

## 3. Results

### 3.1. Detection of anti-MDA5 antibody in JIIM patient

**Figure 1.**
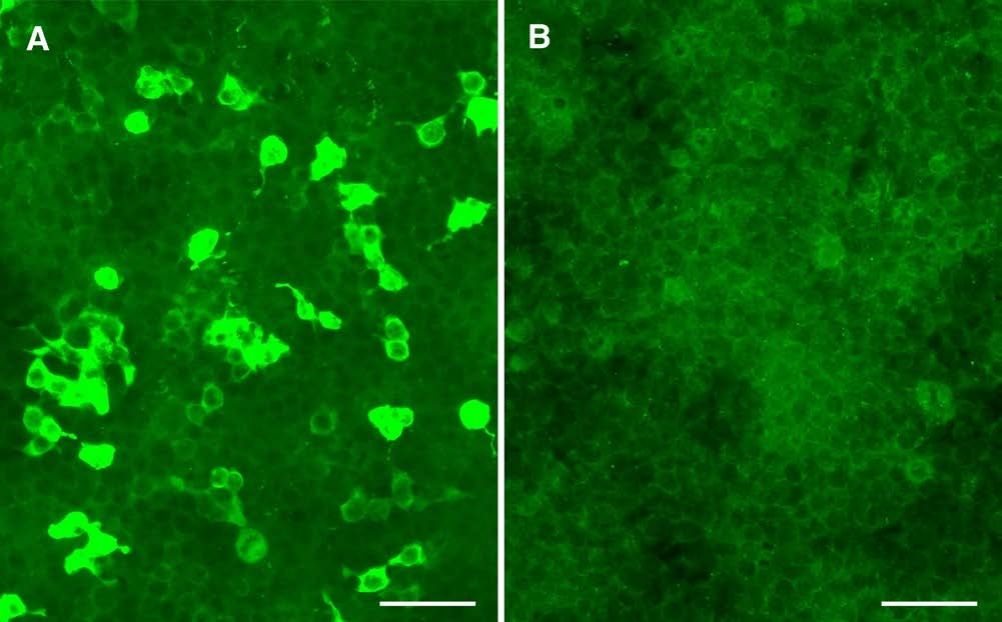
Detection of anti-MDA5 antibodies. (A) IIFA pattern of anti-MDA5 antibody on HEK293 cells showed a membrane fluorescence staining in patient 1. (B) Negative control of MDA5-non-expressing cells in anti-MDA5 antibody-negative JIIM patient. Scale bar = 100 μm. IIFA = indirect immunofluorescence assay; MDA5 = melanoma differentiation associated gene 5.

Anti-MDA5 antibody was detected in 3 (5.08%) of 59 JIIM patients and was confirmed by IIFA (Fig. 1). There were 17 adult IIM patients positive for this antibody, therefore the frequency of anti-MDA5 antibody did not show significant difference between adult IIMs and JIIM cohorts (*P* = .720). All HCs were negative for anti-MDA5 antibody.

### 3.2. Clinical features in anti-MDA5-positive JIIM patients

The clinical features of 3 JIIM patients with anti-MDA5 antibody were summarized in Table 1. The age at onset ranged from 4 to 17 years (mean ± SD, 12.33 ± 7.23 years). The duration from disease onset to the first visit were all within 4 months (2.83 ± 1.04 months). Indeed, typical dermatomyositis skin rashes were seen in all 3 patients. Specifically, 2 patients had Gottron sign and 2 had Helitrope sign. Besides, ILD (not RP-ILD), severe proximal limb weakness, fever, cardiac involvement, arthritis, oral ulceration, and Raynaud phenomenon were seen in 1 patient, respectively. Other extramuscular manifestations such as malignancy and skin calcification were not found in these patients.

**Table 1:** Clinical features and laboratory findings of 3 anti-MDA5-positive JIIM patients.

Patient	Sex/age (year)/disease duration (month)	Muscle symptoms	Extramuscular symptoms	MMT of the weakest muscle group	CK (U/L)	Other MSAs/MAAs	CRP (mg/L)
1	F/4/2.5	PW	Gottron sign, helitrope sign, ILD, oral ulceration and Raynaud phenomenon	4	113	Anti-Ro52	32
2	M/17/2	Myalgia	Helitrope sign, arthritis	5	93	None	9
3	M/16/4	PW, NW, DW, dysphagia	Gottron sign, fever, cardiac involvement	3	72	Anti-pm-scl 75	62

CK = creatine kinase, CRP = C-reactive protein, DW = distal weakness, F = female, ILD = interstitial lung disease, JIIM = idiopathic inflammatory myopathy, M = male, MAA = myositis-associated antibody, MDA5 = melanoma differentiation associated gene 5, MMT = manual muscle testing, MSA = myositis-specific myositis, NW = neck weakness, PW = proximal weakness.

**Figure 2.**
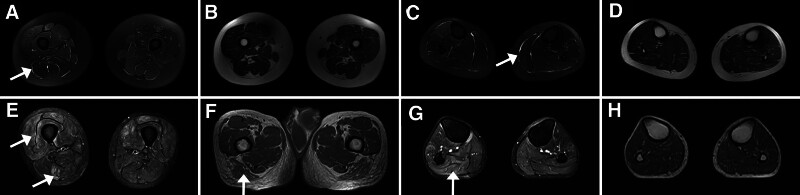
MRI changes in anti-MDA5-positive JIIMs. (A–D) Hyperintense STIR signal in fascia (white arrows) with normal T1 signal of thighs and lower legs in Patient 2. (E) Hyperintense STIR signal (white arrows) in anterior and posterior part of thighs in Patient 3. (F) Hyperintense T1 signal (white arrow) in posterior part of thighs in Patient 3. (G and H) Hyperintense STIR signal (white arrow) without hyperintense T1 signal of posterior lower legs in Patient 3. JIIM = juvenile idiopathic inflammatory myopathy; MDA5 = melanoma differentiation associated gene 5; MRI = magnetic resonance imaging; STIR = short-tau inversion recovery.

The CK levels in serum were normal in all 3 JIIM patients with anti-MDA5 antibody (92.67 ± 20.50 U/L). Besides, we detected anti-Ro52 antibody in patient 1 and anti-PM-Scl75 antibody in patient 3. All 3 patients had high CRP level. Muscle magnetic resonance imaging (MRI) was performed in 2 patients. One showed obvious hyperintense short-tau inversion recovery (STIR) signal in fascia of thighs and lower legs, while another had severe muscle damage with hyperintense STIR signal in both anterior and posterior part and hyperintense T1 signal in posterior part of thighs as well as hyperintense STIR signal without hyperintense T1 signal in posterior lower legs (Fig. 2).

### 3.3. Histopathological findings of muscle biopsies in anti-MDA 5-positive JIIM patients

**Figure 3.**
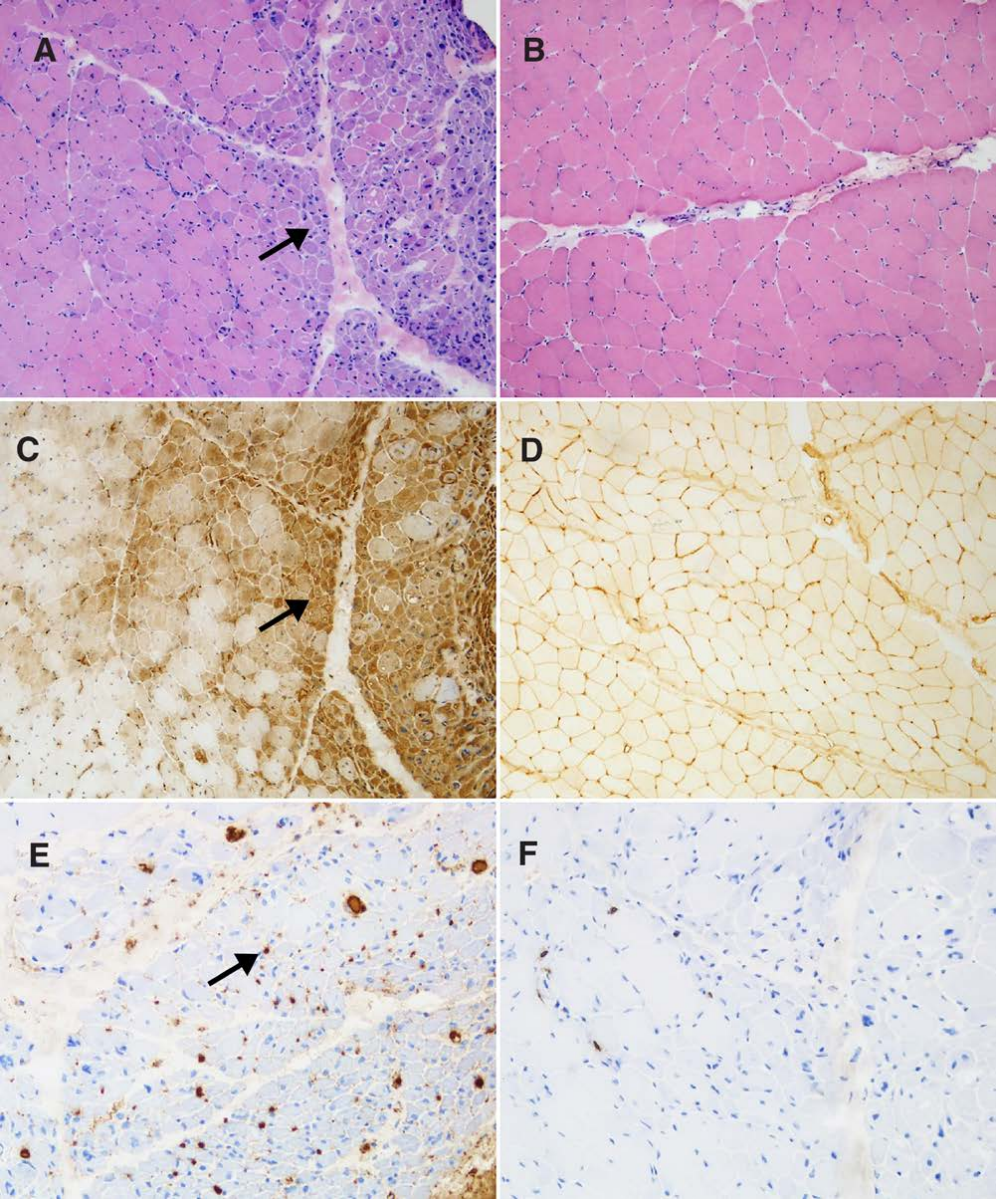
Muscle pathology of anti-MDA5-positive JIIMs. (A) Perifascicular atrophy in Patient 3 (black arrow). (B) Normal HE staining in Patient 2. (C) Perifascicular patterns of MxA expression in Patient 3 (black arrow). (D) Diffuse MHC-I expression in Patient 2. (E) MAC deposition in endomysial capillaries in Patient 3 (black arrow). (F) Few lymphocytic infiltrates in Patient 3. Scale bars = 100 μm. HE = hematoxylin and eosin; JIIM = juvenile idiopathic inflammatory myopathy; MAC = membrane attack complex; MDA5 = melanoma differentiation associated gene 5; MHC-I = major histocompatibility complex class-I; MHC-II = major histocompatibility complex class-II; MxA = myxovirus-resistance protein A.

The histopathological features of 3 JIIM patients with anti-MDA5 antibody were summarized in Table 2. On muscle biopsy, only 1 patient showed obvious myofiber necrosis and regeneration. PF atrophy was found in 1 patient, and MxA expressed in 2 patients including 1 with PF pattern and another with patchy pattern. Besides, all 3 patients had diffuse MHC-I expression and 1 showed MAC-positive staining on endomysial capillaries. PF necrosis, lymphocytic infiltration, MAC deposition in sarcolemma of non-necrotic muscle fibers and MHC-II expression were not detected (Fig. 3).

**Table 2:** Histopathological features of 3 anti-MDA5-positive JIIM patients.

Patient	Inflammation	PA	PN	MxA expression	MHC-I expression	MHC-II expression	MAC capillary/sarcolemmal
1	−	−	−	Focal	Diffuse	−	−/−
2	−	−	−	−	Diffuse	−	−/−
							
3	−	+	−	Perifascicular	Diffuse	−	+/−

JIIM = idiopathic inflammatory myopathy, MAC = membrane attack complex, MDA5 = melanoma differentiation associated gene 5, MHC-I = major histocompatibility complex class-I, MHC-II = major histocompatibility complex class-II, MxA = myxovirus-resistance protein A, PA = perifascicular atrophy, PN = perifascicular necrosis.

### 3.4. Treatment and follow-up of anti-MDA5-positive JIIM patients

In our study, all 3 JIIM patients with anti-MDA5 were followed up for more than 2 years (6.42 ± 4.01 years). The detailed drug therapy and treatment outcomes were listed in Table 3. These patients were all initially treated with oral prednisone (1 mg/kg/day), and subsequently received additional immunotherapies including intravenous immunoglobulin, cyclosporin A, methotrexate or tacrolimus. All 3 patients were medicine free and no 1 suffered from relapse. Additionally, they all showed favorable treatment outcomes as 1 case showed marked improvement and another 2 returned to based line strength.

**Table 3:** Drug therapy and outcomes of 3 anti-MDA5-positive JIIM patients.

Patient	Clinical course (year)	Treatment	Relapse	Drugs at the last follow-up	Outcome
1	10.12	Pred, Cyclosporin A, methotrexate	No	Medicine free	Returning to baseline
2	6.45	Pred, tacrolimus	No	Medicine free	Marked improvement
3	2.40	Pred, IVIG, tacrolimus	No	Medicine free	Returning to baseline

IVIG = intravenous immunoglobulin, JIIM = idiopathic inflammatory myopathy, MDA5 = melanoma differentiation associated gene 5, Pred = prednisone.

## 4. Discussion

This is the first study to evaluate the clinical and histological features as well as treatment outcomes of anti-MDA5 antibody in a large Chinese JIIM cohort. We find that anti-MDA5 antibody is not common in JIIM cases. These anti-MDA5-positive JIIM patients tend to demonstrate increased MHC-I expression in muscles and favorable treatment outcomes without ILD domination.

The prevalence of anti-MDA5 antibodies in our JIIM cohort is 5.08%, which is the lowest compared with other cohorts (about 8–30%).^[6,9,14,23–26]^ Based on the good consistency of findings between immunoblot and IIFA, we consider this low prevalence credible. This can be partly explained by ethnic background as different anti-MDA 5 antibody frequencies were found among races in America.^[27]^

In our JIIM cohort, all anti-MDA5 antibody-positive patients demonstrate typical dermatomyositis (DM) skin lesions including Gottron sign and helitrope sign as previous reports and 2 out of 3 patients had MxA expression.^[9,28]^ Since activated interferon-1 (IFN-1) pathway plays a key role in pathogenesis of DM, the expression of MxA, one of the IFN-1-induced proteins, is regarded as typical DM pathological features to differentiate DM from other diseases.^[29]^ There was a debate about whether anti-MDA5-positive myositis belongs to DM as the former typically have RP-ILD without obvious muscle symptoms.^[19]^ That all our anti-MDA5-positive JIIMs had typical DM rashes and most showed MxA expression supports the view that anti-MDA5-positive myositis should be regarded as a subtype of DM rather than as a separate disease. Besides, the recent finding that anti-MDA5 antibody-positive JIIM showed upregulation of proteins involved in the IFN-1 in serum also supports our viewpoint.^[30]^

Indeed, severe lung involvement, the distinct feature in adult anti-MDA5 DM, is not found in our JIIM patients. It may be due to the fact that anti-Ro52 autoantibody, being proposed as a prognostic marker for ILD in JIIM,^[24]^ is not common in our anti-MDA5-positive JIIMs as well. Unlike adult cases,^[3,31]^ anti-MDA5-positive JIIMs in our study show variable muscle involvement from being amyopathic to severe muscle weakness. This finding was confirmed by muscle MRI and muscle biopsy. Specifically, MRI varies from only fascia edema to obvious edema and fatty pattern in limbs. Muscle biopsy findings also ranges from minimal findings to severe necrosis, though the CK levels are all normal. Moreover, some extramuscular symptoms such as malignance, skin calcification and skin ulceration are absent. As these features have been identified in other anti-MDA5 JIIM cohorts,^[8,14,27,32]^ further studies recruiting larger number of cases are obviously needed. Similar to previous researches, though JIIM with anti-MDA5 antibody showed little or no inflammation, increased MHC-I expression is commonly found and indicates the nature of autoimmunity of anti-MDA5-positive JIIM.^[25,33]^

Our satisfied long-term treatment finding was similar to that reported in previous studies. Unlike anti-MDA5-positive adult IIMs with poor prognosis, our JIIM patients show remarkably improvement of motor functions without relapse, though additional immunotherapies besides prednisone are required as reported.^[8,9,11,14,26,34]^ This favorable outcome is partly due to the absence of RP-ILD and other severe complications.^[19,24,35]^ As Ichiro Kobayashi and colleagues reported that anti-MDA5-positive JIIM patients with ILD could achieve complete recovery and maintain a drug-free status, the patient with ILD (P1) also received a favorable outcome.^[10,14]^ Therefore, we indicated that anti-MDA5-positive JIIM cases without RP-ILD could obtain a favorable prognosis.

The limitation of the present study lies in the small sample size of anti-MDA5-positive JIIM patients and this limited the comparisons among anti-MDA5-positive JIIMs and other MSA-positive ones. Further studies, in particular comparative studies with large sample size, are required to confirm the clinical, histological features and treatment outcomes of anti-MDA5-positive JIIMs.

## 5. Conclusions

Anti-MDA5 antibodies are not frequently found in Chinese JIIM patient. It delineates a group of JIIM patients with typical DM rashes, increased MxA and MHC-I expression, normal CK level without ILD predominance. Multi-immunotherapy generally leads to favorable outcome. We suggest that anti-MDA5 antibody should be routinely tested in JIIM for disease prognosis. Given that our present study is a retrospective review of a relatively small sample of anti-MDA5-positive JIIM, further studies based on larger cohorts of patients are required to support our conclusions.

## Acknowledgments

The authors thank the patients and controls for their participations in this study.

## Author contributions

**Conceptualization:** Ying Hou, Dandan Zhao.

**Data curation:** Ying Hou.

**Supervision:** Dandan Zhao.

**Validation:** Dandan Zhao.

**Visualization:** Dandan Zhao.

**Writing – original draft:** Long Liu.

**Writing – review & editing:** Dandan Zhao.

## References

[1] Dalakas MC. Inflammatory muscle diseases. N Engl J Med. 2015;372:1734–47. 25923553 10.1056/NEJMra1402225

[2] Lundberg IE Tjarnlund A Bottai M. 2017 European League Against Rheumatism/American College of Rheumatology classification criteria for adult and juvenile idiopathic inflammatory myopathies and their major subgroups. Ann Rheum Dis. 2017;76:1955–64. 29079590 10.1136/annrheumdis-2017-211468 PMC5736307

[3] McHugh NJ Tansley SL. Autoantibodies in myositis. Nat Rev Rheumatol. 2018;14:290–302. 29674612 10.1038/nrrheum.2018.56

[4] Papadopoulou C Chew C Wilkinson MGL McCann L Wedderburn LR. Juvenile idiopathic inflammatory myositis: an update on pathophysiology and clinical care. Nat Rev Rheumatol. 2023;19:343–62. 37188756 10.1038/s41584-023-00967-9 PMC10184643

[5] Mamyrova G Kishi T Targoff IN Ehrlich A Curiel RV Rider LG. Features distinguishing clinically amyopathic juvenile dermatomyositis from juvenile dermatomyositis. Rheumatology (Oxford). 2018;57:1956–63. 30016492 10.1093/rheumatology/key190 PMC6199536

[6] Gupta L Naveen R Gaur P Agarwal V Aggarwal R. Myositis-specific and myositis-associated autoantibodies in a large Indian cohort of inflammatory myositis. Semin Arthritis Rheum. 2021;51:113–20. 33360322 10.1016/j.semarthrit.2020.10.014

[7] Sato S Hoshino K Satoh T. RNA helicase encoded by melanoma differentiation-associated gene 5 is a major autoantigen in patients with clinically amyopathic dermatomyositis: Association with rapidly progressive interstitial lung disease. Arthritis Rheum. 2009;60:2193–200. 19565506 10.1002/art.24621

[8] Tansley SL Betteridge ZE Gunawardena H. Anti-MDA5 autoantibodies in juvenile dermatomyositis identify a distinct clinical phenotype: a prospective cohort study. Arthritis Res Ther. 2014;16:R138. 24989778 10.1186/ar4600 PMC4227127

[9] Mamyrova G Kishi T Shi M. Anti-MDA5 autoantibodies associated with juvenile dermatomyositis constitute a distinct phenotype in North America. Rheumatology (Oxford). 2021;60:1839–49. 33140079 10.1093/rheumatology/keaa429 PMC8023991

[10] Kobayashi I Okura Y Yamada M Kawamura N Kuwana M Ariga T. Anti-melanoma differentiation-associated gene 5 antibody is a diagnostic and predictive marker for interstitial lung diseases associated with juvenile dermatomyositis. J Pediatr. 2011;158:675–7. 21232756 10.1016/j.jpeds.2010.11.033

[11] Hussain A Rawat A Jindal AK Gupta A Singh S. Autoantibodies in children with juvenile dermatomyositis: a single centre experience from North-West India. Rheumatol Int. 2017;37:807–12. 28331982 10.1007/s00296-017-3707-4

[12] Ueda-Hayakawa I Tonomura K Maekawa A Kaneda E Arase N Fujimoto M. Age distribution and prevalence in different age groups of four myositis-specific autoantibodies, including anti-ARS, anti-MDA5, anti-Mi-2, and anti-TIF1gamma antibodies. J Dermatol. 2023;50:1058–62. 36890683 10.1111/1346-8138.16772

[13] Horn S Minden K Speth F. Myositis-specific autoantibodies and their associated phenotypes in juvenile dermatomyositis: data from a German cohort. Clin Exp Rheumatol. 2022;40:433–42. 33124555 10.55563/clinexprheumatol/94btoy

[14] Ueki M Kobayashi I Takezaki S. Myositis-specific autoantibodies in Japanese patients with juvenile idiopathic inflammatory myopathies. Mod Rheumatol. 2019;29:351–6. 29532710 10.1080/14397595.2018.1452353

[15] Soponkanaporn S Deakin CT Schutz PW. Expression of myxovirus-resistance protein A: a possible marker of muscle disease activity and autoantibody specificities in juvenile dermatomyositis. Neuropathol Appl Neurobiol. 2019;45:410–20. 29770465 10.1111/nan.12498 PMC6563435

[16] Hou C Durrleman C Periou B. From diagnosis to prognosis: revisiting the meaning of muscle ISG15 overexpression in juvenile inflammatory myopathies. Arthritis Rheumatol. 2021;73:1044–52. 33314705 10.1002/art.41625

[17] Tanboon J Inoue M Saito Y. Dermatomyositis: muscle pathology according to antibody subtypes. Neurology. 2021;98:e739–e749. 34873015 10.1212/WNL.0000000000013176 PMC8865893

[18] Hoogendijk JE Amato AA Lecky BR. 119th ENMC international workshop: trial design in adult idiopathic inflammatory myopathies, with the exception of inclusion body myositis, 10-12 October 2003, Naarden, The Netherlands. Neuromuscul Disord. 2004;14:337–45. 15099594 10.1016/j.nmd.2004.02.006

[19] Mammen AL Allenbach Y Stenzel W Benveniste O. 239th ENMC International Workshop: classification of dermatomyositis, Amsterdam, the Netherlands, 14-16 December 2018. Neuromuscul Disord. 2020;30:70–92. 31791867 10.1016/j.nmd.2019.10.005

[20] Kobayashi N Takezaki S Kobayashi I. Clinical and laboratory features of fatal rapidly progressive interstitial lung disease associated with juvenile dermatomyositis. Rheumatology (Oxford). 2015;54:784–91. 25288783 10.1093/rheumatology/keu385

[21] Kassardjian CD Lennon VA Alfugham NB Mahler M Milone M. Clinical features and treatment outcomes of necrotizing autoimmune myopathy. JAMA Neurol. 2015;72:996–1003. 26192196 10.1001/jamaneurol.2015.1207

[22] Hou Y Shao K Yan Y. Anti-HMGCR myopathy overlaps with dermatomyositis-like rash: a distinct subtype of idiopathic inflammatory myopathy. J Neurol. 2021;269:280–93. 34021410 10.1007/s00415-021-10621-7

[23] Sag E Demir S Bilginer Y. Clinical features, muscle biopsy scores, myositis specific antibody profiles and outcome in juvenile dermatomyositis. Semin Arthritis Rheum. 2021;51:95–100. 33360233 10.1016/j.semarthrit.2020.10.007

[24] Sabbagh S Pinal-Fernandez I Kishi T. Anti-Ro52 autoantibodies are associated with interstitial lung disease and more severe disease in patients with juvenile myositis. Ann Rheum Dis. 2019;78:988–95. 31018961 10.1136/annrheumdis-2018-215004 PMC7570952

[25] Yasin SA Schutz PW Deakin CT. Histological heterogeneity in a large clinical cohort of juvenile idiopathic inflammatory myopathy: analysis by myositis autoantibody and pathological features. Neuropathol Appl Neurobiol. 2019;45:495–512. 30378704 10.1111/nan.12528 PMC6767402

[26] Yamasaki Y Kobayashi N Akioka S. Clinical impact of myositis-specific autoantibodies on long-term prognosis of juvenile idiopathic inflammatory myopathies: multicentre study. Rheumatology (Oxford). 2021;60:4821–31. 33576399 10.1093/rheumatology/keab108

[27] Perron MM Vasquez-Canizares N Tarshish G Wahezi DM. Myositis autoantibodies in a racially diverse population of children with idiopathic inflammatory myopathies. Pediatr Rheumatol Online J. 2021;19:92. 34118936 10.1186/s12969-021-00574-6 PMC8199392

[28] Iwata N Nakaseko H Kohagura T. Clinical subsets of juvenile dermatomyositis classified by myositis-specific autoantibodies: experience at a single center in Japan. Mod Rheumatol. 2019;29:802–7. 30092736 10.1080/14397595.2018.1511025

[29] Greenberg SA Pinkus JL Pinkus GS. Interferon-alpha/beta-mediated innate immune mechanisms in dermatomyositis. Ann Neurol. 2005;57:664–78. 15852401 10.1002/ana.20464

[30] Sato H Inoue Y Kawashima Y. In-depth proteomic analysis of juvenile dermatomyositis serum reveals protein expression associated with muscle-specific autoantibodies. Rheumatology (Oxford). 2023;62:3501–6. 37052527 10.1093/rheumatology/kead165

[31] Abe Y Matsushita M Tada K Yamaji K Takasaki Y Tamura N. Clinical characteristics and change in the antibody titres of patients with anti-MDA5 antibody-positive inflammatory myositis. Rheumatology (Oxford). 2017;56:1492–7. 28499006 10.1093/rheumatology/kex188

[32] Tansley SL Simou S Shaddick G. Autoantibodies in juvenile-onset myositis: Their diagnostic value and associated clinical phenotype in a large UK cohort. J Autoimmun. 2017;84:55–64. 28663002 10.1016/j.jaut.2017.06.007 PMC5656106

[33] Melki I Devilliers H Gitiaux C. Anti-MDA5 juvenile idiopathic inflammatory myopathy: a specific subgroup defined by differentially enhanced interferon-alpha signalling. Rheumatology (Oxford). 2020;59:1927–37. 31755959 10.1093/rheumatology/kez525

[34] Dunga SK Kavadichanda C Gupta L Naveen R Agarwal V Negi VS. Disease characteristics and clinical outcomes of adults and children with anti-MDA-5 antibody-associated myositis: a prospective observational bicentric study. Rheumatol Int. 2021;42:1155–65. 34050793 10.1007/s00296-021-04897-1

[35] Ma X Chen Z Hu W. Clinical and serological features of patients with dermatomyositis complicated by spontaneous pneumomediastinum. Clin Rheumatol. 2016;35:489–93. 26149923 10.1007/s10067-015-3001-3

